# Supplementary material for: Pseudomonas aeruginosa Leucine Aminopeptidase Influences Early Biofilm Composition and Structure via Vesicle-Associated Antibiofilm Activity
Source: mBio. 2019 Nov 19;10(6):e02548-19. doi: 10.1128/mBio.02548-19 (PMC6867898; doi:10.1128/mBio.02548-19)
Supplement: TABLE S1 [file mBio.02548-19-st001.pdf]

Table S1: qRT-PCR primers used in this study

qRT-PCR primers		
**Target**	**Sequence (5'-3')**	
proC[1]	F	CAGGCCGGGCAGTTGCTGTC
	R	GGTCAGGCGCGAGGCTGTCT
16s rRNA[2]	F	CAAAACTACTGAGCTAGAGTACG
	R	TAAGATCTCAAGGATCCCAACGGCT
PaAP[3]	F	GTGGTACGCAAGAAGACCGA
	R	ATCACCACGTTGTTCGGGTT
FliD[4]	F	CCAGTTCAAGAGTGCGATCA
	R	ACGGAGATGTTCAGCGTACC
CupA1[5]	F	GCGGCAAACACTATCACATTC
	R	AACAGGGTGGTGAAATGCTC
PilA[5]	F	AACCTGAACCTGGACTGTGG
	R	TTGCCTTCGCCATCTTTT
PslA[6]	F	AAGATCAAGAAACGCGTGGAAT
	R	TGTAGAGGTCGAACCACACCG

References

1. Savli, H. *et al.* Expression stability of six housekeeping genes: A proposal for resistance gene quantification studies of Pseudomonas aeruginosa by real-time quantitative RT-PCR. *Journal of medical microbiology* **52,** 403–8 (2003).

2. Lenz, A. P., Williamson, K. S., Pitts, B., Stewart, P. S. & Franklin, M. J. Localized Gene Expression in Pseudomonas aeruginosa Biofilms ▿ . *Appl Environ Microb* **74,** 4463–4471 (2008).

3. Mellbye, B. & Schuster, M. Physiological Framework for the Regulation of Quorum Sensing-Dependent Public Goods in Pseudomonas aeruginosa. *Journal of Bacteriology* **196,** 1155–1164 (2014).

4. Deng, X. *et al.* Steady-State Hydrogen Peroxide Induces Glycolysis in Staphylococcus aureus and Pseudomonas aeruginosa. *J Bacteriol* **196,** 2499–2513 (2014).

5. Qaisar, U. *et al.* The pvc Operon Regulates the Expression of the Pseudomonas aeruginosa Fimbrial Chaperone/Usher Pathway (Cup) Genes. *Plos One* **8,** e62735 (2013).

6. Colvin, K. M. *et al.* The Pel and Psl polysaccharides provide Pseudomonas aeruginosa structural redundancy within the biofilm matrix. *Environ Microbiol* **14,** 1913–1928 (2012).
